# Luminescence Characteristics of the MOCVD GaN Structures with Chemically Etched Surfaces

**DOI:** 10.3390/ma16093424

**Published:** 2023-04-27

**Authors:** Tomas Ceponis, Jevgenij Pavlov, Arunas Kadys, Augustas Vaitkevicius, Eugenijus Gaubas

**Affiliations:** Institute of Photonics and Nanotechnology, Vilnius University, Sauletekio Ave. 3, LT-10257 Vilnius, Lithuania; jevgenij.pavlov@tmi.vu.lt (J.P.); arunas.kadys@ff.vu.lt (A.K.); augustas.vaitkevicius@ff.vu.lt (A.V.); eugenijus.gaubas@ff.vu.lt (E.G.)

**Keywords:** gallium nitride, dislocations, wet etching, photoluminescence, pulsed photo-ionization spectroscopy

## Abstract

Gallium nitride is a wide-direct-bandgap semiconductor suitable for the creation of modern optoelectronic devices and radiation tolerant detectors. However, formation of dislocations is inevitable in MOCVD GaN materials. Dislocations serve as accumulators of point defects within space charge regions covering cores of dislocations. Space charge regions also may act as local volumes of enhanced non-radiative recombination, deteriorating the photoluminescence efficiency. Surface etching has appeared to be an efficient means to increase the photoluminescence yield from MOCVD GaN materials. This work aimed to improve the scintillation characteristics of MOCVD GaN by a wet etching method. An additional blue photo-luminescence (B-PL) band peaking at 2.7–2.9 eV and related to dislocations was discovered. This B-PL band intensity appeared to be dependent on wet etching exposure. The intensity of the B-PL was considerably enhanced when recorded at rather low temperatures. This finding resembles PL thermal quenching of B-PL centers. The mechanisms of scintillation intensity and spectrum variations were examined by coordinating the complementary photo-ionization and PL spectroscopy techniques. Analysis of dislocation etch pits was additionally performed by scanning techniques, such as confocal and atomic force microscopy. It was proved that this blue luminescence band, which peaked at 2.7–2.9 eV, is related to point defects those decorate dislocation cores. It was shown that the intensity of this blue PL band was increased due to enhancement of light extraction efficiency, dependent on the surface area of either single etch-pit or total etched crystal surface.

## 1. Introduction

Gallium nitride (GaN) is a wide-direct-bandgap semiconductor suitable for the creation of modern optoelectronic devices and radiation-tolerant detectors [1,2,3,4,5,6]. This material can be synthesized using various crystal growth methods [7]. Metalorganic chemical vapor deposition (MOCVD) is commonly used for growing of rather thin GaN crystal layers on various substrates. However, the density of dislocations in these crystals can reach 10^10^ cm^−2^ [8,9], while small density of vacancies is inherent to MOCVD materials [10]. Modern growth methods of bulk GaN crystals, such as ammonothermal (AT), hydride vapor phase epitaxy (HVPE), and lateral epitaxial overgrowth (LEO) techniques, enable reduction in the density of dislocations to electronic grade values of 100 cm^−2^ [11,12]. Nevertheless, a rather high density of voids is inevitable for AT materials [13,14,15]. Edge, screw, and mixed-type dislocations serve as accumulators of point defects within space charge regions covering cores of dislocations. Space charge regions act as local volumes of enhanced recombination and reduced carrier mobility [16,17]. The outspreading of space charge regions also depends on free carrier density in GaN crystals doped with various impurities, such as Si, Mg, etc. In MOCVD GaN, these dislocations might compose disordered material nets with inherent carrier transport and recombination features. The disorder might be a reason for considerably delayed signals of photoconductivity and photoluminescence [18]. The disorder might also lead to the stretched exponent relaxation (SER) of excess carriers. Nevertheless, MOCVD GaN can be employed for the fabrication of double response radiation sensors, in which both the electrical and the scintillation signals are simultaneously recorded. Therefore, enhancement of the efficiency of the electrical and scintillation responses of MOCVD GaN materials is desirable. Surface etching appears to be an efficient means to increase the photoluminescence yield from MOCVD GaN materials.

Surface regenerating can be implemented by wet (chemical) etching [9,19,20,21]. GaN crystal surface modification (dry etching) can also be performed using reactive ion plasma etching (RIE) through the creation of nanowires by a maskless lithography process [22,23,24,25,26]. Light extraction efficiency (LEE) from dislocation-rich GaN scintillators is limited by internal reflection and polarization factors [2,3,6]. LEE can be enhanced by modifying the geometry of the crystal surface and its effective area. Wet etching leads to the formation of pits due to different etching rates assigned to various crystalline planes and types of dislocations [19,20,21]. Macroscopic polarization is inherent to GaN and its alloys due to spontaneous and piezoelectric polarization, leading to modifications of light extraction efficiency dependent on local strains. Local stress can also be relieved by increasing the density and size of the etching pits [21]. Thereby, wet and dry etching can also cause changes in the photoluminescence characteristics of GaN crystals [27,28,29,30].

This work aimed to improve the scintillation characteristics of MOCVD GaN double response sensors using wet etching techniques. Defects responsible for luminescence spectrum variations were controlled by coordinating the complementary photo-ionization and PL spectroscopy techniques. An additional blue luminescence band peaking at 2.7–2.9 eV and dependent on wet etching exposure was revealed. The intensity of this PL band was considerably enhanced when scintillation was recorded at rather low temperatures. Localization of the radiative recombination centers responsible for this blue PL band was identified by combining scanning techniques, such as confocal and atomic force microscopy. The origins of the prevailing radiative recombination centers were separated by employing different excitation wavelengths, namely 354-nm pulsed laser beams suitable to efficiently stimulate electronic transitions within C_N_O_N_ complexes and 408-nm laser diode illumination to activate the C_N_ centers, according to configuration diagram models presented in Ref. [31]. The hydrogen complexes with carbon-originated point defects appeared to be the non-homogeneously distributed blue luminescence centers those decorate cores of dislocations. These B-PL centers appeared to be sensitive to thermal quenching. It was shown that the intensity of this blue PL band was increased due to enhancement of light extraction efficiency, dependent on the surface area of either single etch-pits or the total etched crystal surface. The etch-modified surface area increases with the duration of the etching process and the temperature of the etchant. Thereby, etching technologies can serve as tools to govern the scintillation properties of MOCVD grown GaN crystals and devices.

## 2. Samples and Measurement Techniques

In this work, 3.8 μm-thick GaN epi-layers grown on sapphire substrates were examined. These GaN epi-layers were grown using an MOCVD closed-coupled showerhead reactor. The 430 ± 25 μm-thick sapphire substrate with surface roughness of <0.2 nm was on a c-plane inclined by about 0.20 degrees in the m-plane direction. Trimethylgallium (TMG), silane (SiH_4_), and ammonia (NH_3_) were used as Ga, Si, and N precursors, respectively. The processes from sapphire surface pre-treatment to the growth of the final Si-doped GaN layer were conducted in a hydrogen atmosphere. The 0.9 μm-thick buffer layer of un-doped GaN (u-GaN) was initially deposited. The functional GaN was grown at a temperature of *T*_gr_ = 1040 °C and an mbar pressure of *p* = 100. This functional GaN layer was doped with Si of a nominal concentration of *N_Si_* ≅ 10^17^ cm^−3^. The free electron concentration of 10^17^ cm^−3^ and mobility of up to 430 cm^2^/V·s were determined by Hall effect measurements at room temperature. Concentrations of carbon impurities were estimated to be *N_C_* > 10^16^ cm^−3^ in MOCVD GaN layers grown using a similar regime. The photoluminescence (PL) spectra and scintillation intensity topography were initially examined. Rather homogeneous samples (of 6 × 6 mm^2^ area) concerning scintillation spectrum structure and intensity were cut from MOCVD GaN-layered wafers. Values of PL intensity within yellow-green spectral band peaks were further exploited for normalization of spectral and intensity changes dependent on the temperature and etching regimes. The examined MOCVD GaN layers were etched using orthophosphoric acid (85% H_3_PO_4_), varying its temperature in the range of 90–160 °C. The etch exposure *t_i_* was varied in the range of 0–1600 s in increasing duration of this procedure by 200 s steps (*i* = 0–8). Thereby, a collection of samples created using different temperatures and exposure parameters was examined. Additionally, a single sample that aggregated a set of etching exposures was also controlled after each etching procedure.

Scanning electron microscope (SEM) imaging was initially employed for etch-pit control after short etching exposures. An Olympus BX51 microscope (Olympus Corporation, Tokyo, Japan) was used for the preliminary inspection of etched layers under ultraviolet (UV) light illumination. The average density of the dislocations of 8 × 10^8^ cm^−2^ was extracted by calculating the etch-pits within definite areas of SEM images and including all dislocation types. Profiling of dislocation etching pits was implemented using atomic force microscope (AFM) imaging performed by a WITEC microscopy system Alpha 300 (WITec GmbH, Ulm, Germany). The latter AFM system enabled etch-pit profiling with vertical and in-plane resolutions of 1 and 10 nm, respectively. AFM scans were used to estimate the density of dislocations within etched MOCVD GaN layer fragments and to identify the types of prevailing dislocations using etch-pit shape analysis.

Photoluminescence spectroscopy (PL) and topography were performed under steady-state and pulsed laser excitation. The pulsed PL spectroscopy measurements were performed with varying sample temperatures in the range of 20–300 K. The closed-cycle He cryogenic system, together with the sample mounting arrangement, was used for temperature-stabilized measurements. Pulsed 400-ps excitation was implemented using a UV 354-nm laser (STA-03 “Standa”) beam. PL spectra were recorded by accumulating and averaging hundreds of PL response pulses within an Avantes AvaSpec-ULS2048XL-EVO spectrophotometer (Avantes B.V., Apeldoorn, The Netherlands). These measurements were combined with room-temperature PL spectroscopy correlated with etched surface topography. The latter PL measurements were performed using the multi-functional WITEC system Alpha 300 (WITec GmbH, Ulm, Germany), implemented in confocal microscopy mode. The PL light signal captured within the confocal image was transmitted via proper optical fiber to a thermoelectrically cooled CCD camera and a UHT300 spectrometer. A continuous-wave (CW) laser diode emitting at a fixed wavelength of 405 nm was there for PL excitation. A high numerical aperture (NA = 0.9) objective was applied to focus the excitation beam on the sample, providing an in-plane spatial resolution of around 250 nm and a vertical resolution of about 1000 nm. Different excitation wavelengths were combined to clarify the origins of the prevailing radiative recombination centers. The 354-nm pulsed laser beams were suitable to efficiently stimulate electronic transitions within C_N_O_N_ complexes, while 408-nm laser diode illumination can be employed to activate the C_N_ centers, according to configuration diagram models presented in Ref. [31]. The correlated PL and photoconductivity transients were additionally examined to evaluate the temporal parameters of pulsed signals. Additionally, pulsed photo-ionization spectroscopy (PPIS) was employed to correlate Stokes shifts between excitation and PL spectral bands ascribed to the same scintillation centers. The PPIS method was implemented at varying excitation photon wavelengths in the range of 210–2300 nm generated by a 4-ns Ekspla NT342B Optical Parametric Oscillator (OPO) instrument (Ekspla UAB, Vilnius, Lithuania). The photon-electron interaction spectral steps were recorded in contactless mode by 20 to 22 GHz microwave probing of the photo-ionized carrier density and its relaxation rate. PPI step-structure spectra appeared due to variations in the MW-PC response amplitudes when the energy of excitation photons was scanned over a rather wide spectral range. A spectral step peak appeared when the photon energy (*hν*) matched the energy between the electron ground state and that of a deep level associated with a defect. The activation energy *E_d_* of photo-absorption due to defective photo-ionization is related to the photon-electron interaction cross-section *σ_ph-e_*. The most comprehensive approach to estimating *σ_ph-e_* is the Kopylov–Pikhtin model [32]. The activation energy *E_d_* of a photoactive center is related to the photon-electron interaction cross-section as follows:

(1) $$\sigma_{ph-e}\left(h\nu \right)=M_{ik}{}^{2}\int_{0}^{\infty }\frac{e^{-{\left(E+E_{d}-hv\right)}^{2}/\Gamma^{2}}\sqrt{E}dE}{hv{\left(E+E_{d}\right)}^{2}}.$$

Here, *M_ik_* is the matrix element of a dipole transition from an initial (*i*) trap level to the continuum (*k*) state, and integration over all the conduction band states *E* is performed. The electron-phonon coupling is also determined by the broadening factor *Γ,* which depends on temperature (thermal energy *k_B_T*) as: $\Gamma =\sqrt{2S}\nu_{0}\sqrt{2\cot h\left(h\nu_{0}/k_{B}T\right)}$. Here, *S* is the Huang–Rhys [32,33] factor. The photon-electron interaction cross-section *σ_ph-e_* is thereby related to the Franck–Condon shift and the energy of the vibrational modes [34]. The cross-section *σ_ph-e_* of the photon-electron coupling directly determines the efficiency of the conversion of absorption into emission [34,35].

## 3. Correlation of PL Spectra with Photo-Ionization Characteristics

**Figure 1:**
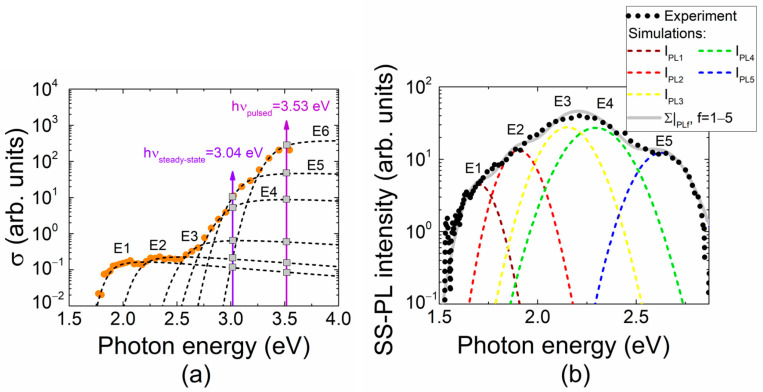
(**a**) The photo-activation centers (E1–E6) resolved from the photo-ionization spectral steps, measured on MOCVD GaN (scattered circles-data) and simulated using Kopylov–Pikhtin [32] approach (Equation (1))-dashed lines. These traps were tentatively identified using the activation energy values taken from the literature referenced. The peak values of spectral bands were adjusted by varying the trap concentration parameters. Arrows denote the photon energy for fixed wavelength excitation of photoluminescence employed in these experiments. The gray rectangles indicate a relative efficiency of PL excitation by intersection of photon energy with spectral shapes of the identified photo-ionization centers. (**b**) PL spectrum (black circle symbols) recorded on MOCVD GaN material at room temperature using the multi-functional WITEC system Alpha 300 in confocal microscopy mode. This measured spectrum was fitted (gray solid line) using the van Roosbroeck–Shockley model (Equation (2)). This simulated gray solid line represents a resultant PL spectrum of simultaneous action of photoactive centers revealed by pulsed photo-ionization spectroscopy (PPIS) techniques. The contribution of each center (dashed lines) was simulated using parameters extracted from PPIS analysis. Fitting of the experimental PL spectrum (scattered circles) by a simulated resultant PL spectrum (solid gray curve) was implemented by slightly adjusting the contribution of various centers (dashed lines). The latter procedure was performed by varying the peak amplitudes (related to concentrations of definite centers).

Photoluminescence excitation in our experiments was performed by UV (354 nm) and violet (408 nm) laser beams, as mentioned above, to separate the prevailing radiative recombination centers. The UV laser beam generates excess carriers through inter-band electron transitions, which later recombine through excitonic annihilation and radiative processes via deep electronic states ascribed to various defects. Together with inter-band processes, the excess carriers can be generated by photo-ionization of defects, leading to a step-like spectrum of absorption. The pulsed photo-ionization spectra (PPIS) recorded at room temperature in MOCVD GaN are illustrated in Figure 1a. Several carrier photo-activation centers (up to six traps for photon energies in the range of 1.0–3.3 eV) are inherent to MOCVD GaN materials. Separation of the PPI spectral steps was accomplished by controlling whether the same step appeared in different samples. Non-resonant excitation was implemented using fixed-wavelength light (354 and 408 nm) illumination with relative efficiency (determined by relative absorption coefficients and denoted in Figure 1a by square points) attributed to various defects. An absorption coefficient *α_d_*(*hν*) = *σ_d_*(*hν*)*n_d_* related to a definite trap also depends on filling of its levels *n_d_*.

The similar structure of the photo-ionization spectra has been revealed [36] for the MOCVD GaN layers grown on Si 2 mm-thick substrates using close growth parameters (*T*_gr_ = 1040 °C, *p* = 100 mbar, *N_C_* ≈ 5 × 10^16^ cm^−3^). The correlation between the photo-ionization spectra obtained for MOCVD GaN layers grown on different substrates (such as Si [36] and sapphire in this work) using similar growth parameters indicates that the same radiative recombination centers prevail.

Figure 1a shows the *σ_d_*(*hν*) spectrum illustrating *α_d_*(*hν*) with normalized *n_d_* distribution inherent for MOCVD GaN materials. It can be deduced from Figure 1 that excess carriers excited by UV laser are capable of inducing photo-luminescence from all the traps (highlighted as the spectral steps) including exciton annihilation, followed by phonon replicas, while the laser diode radiation at 408 nm wavelength affords ionization of the centers with activation energy less than that of the E5 trap. The origin of traps, tentatively identified by coinciding peak values of activation energy referenced in literature, is denoted in Table 1.

Several resolved deep photoactive centers with activation energies in the range of 1.3–3.3 eV indicate a large nomenclature of point defects inherent to MOCVD GaN materials. This large amount of different species of point defects complicates the analysis of each definite center. The more reliable estimation of optically active centers would be fitting to the conversion from absorption to light emission spectra. The van Roosbroeck–Shockley relation [33,34], based on the detail balance condition, is acceptable for describing the spectral shift of light the emission rate ∆*P_d_*(*hν*) and the temperature dependent cross-section *σ*(*hν/k_B_T*) attributed to the *d*-th center [40]:

(2) $$∆P_{d}\left(h\nu \right)=\rho_{d}\left(\frac{h\nu }{k_{B}T}\right)\sigma_{d}(h\nu /k_{B}T)N_{d}\frac{n_{ex,∆\left(h\nu \right)}N_{d}}{n_{i}^{2}}.$$

Here, *n_i_* = 2 × 10^−10^ cm^−3^ is the intrinsic carrier concentration for GaN [41]; *k_B_* is the Boltzmann constant, *h* is the Planck constant; *ρ_d_* is the surface density of photons ascribed to a fixed frequency *ν* within absorption spectra for the spectral range Δ(*hν*), inherent to a dedicated trap of concentration *N_d_*; and *n_ex_*_,Δ(*hν*)_ is the excess carrier density generated through photo-ionization in the definite spectral range Δ(*hν*). This approach enables prediction of the Stokes shifts between the outspread PPIS steps and the respective PL band peaks. The temperature dependence in *σ*(*hν*/*k_B_T*) appears through the broadening factor *Γ*(*hν*/*k_B_T*)*,* which is rather weak in the range of low and moderate temperatures.

**Figure 2:**
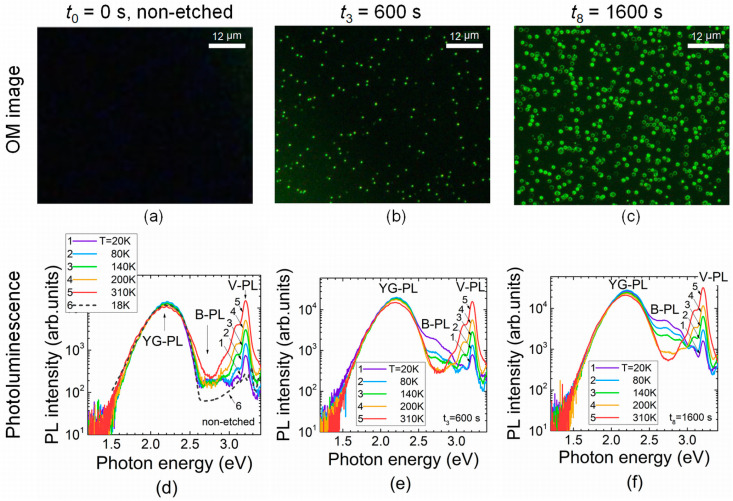
(**a**–**c**) Evolution of optical microscopy (OM) images dependent on etching exposure. (**d**–**f**) PL spectra measured at different sample temperatures and dependent on etching exposure duration (*t*) when an H_3_PO_4_ temperature of 150 °C was kept over etching procedures. A sketch (curve 6) of the PL spectrum typical for the MOCVD grown GaN is taken from Ref. [43] and plotted in Figure (**d**) as a dashed curve for comparison of inherent spectral structures.

A predicted PL spectrum in MOCVD GaN (simulated using photo-ionization spectroscopy data) is illustrated in Figure 1b. The red-shifted (relative to photo-ionization spectral peaks) bands of yellow–green luminescence (YG-PL), composed of the radiative recombination through E_1_–E_4_ traps, and blue (B-PL) luminescence are in line with our experimental observations in photo-ionization and photoluminescence spectra. Here, the YG-PL band is determined by the radiative recombination through E_1_–E_4_ traps, while B-PL appears due to E_5_ traps. The additional recombination channel, observed as a violet PL (V-PL) band (within spectra illustrated in Figure 2d–f), peaked at 3.2–3.3 eV, and it has been traditionally (Ref. [42]) attributed to the donor–acceptor pair recombination.

## 4. Correlation of PL Spectra with Etch–Pit Profiling Scans in MOCVD GaN Layers

**Figure 3:**
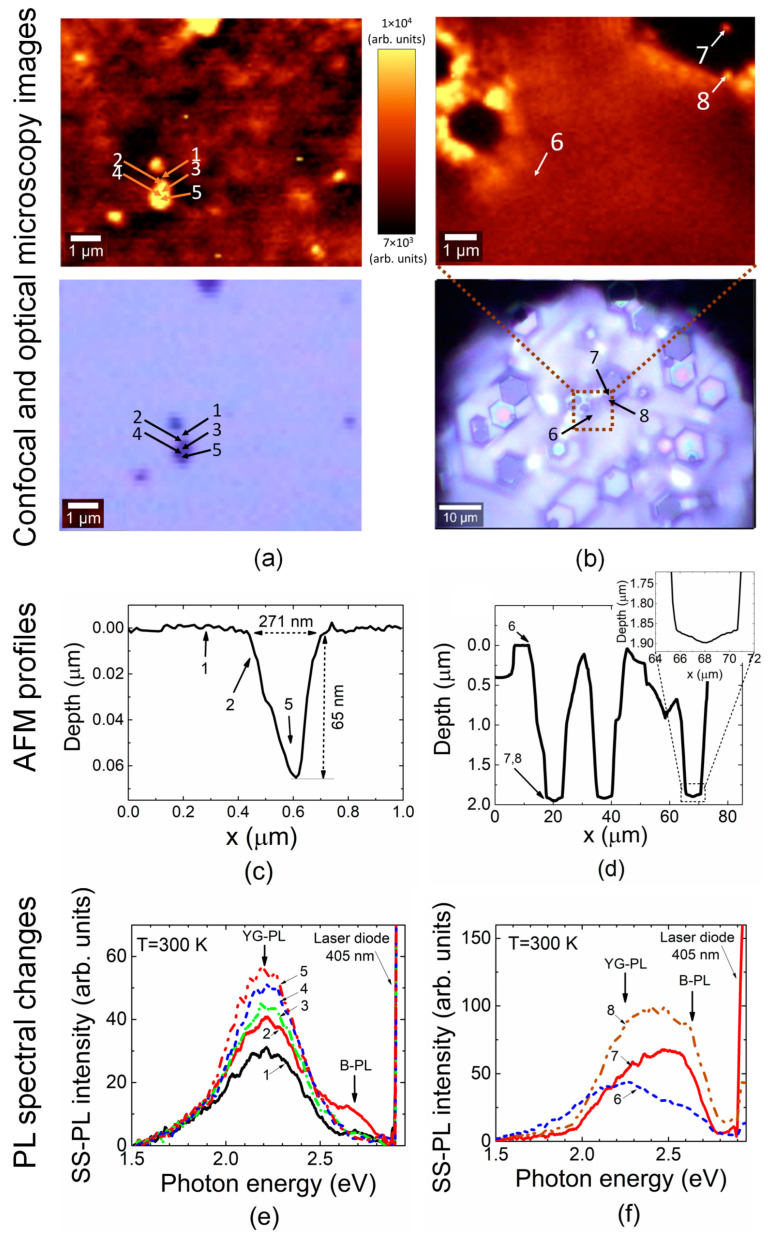
Correlation among confocal microscopy (of PL integrated within 450 to 720 nm spectral range) and optical microscopy images (**a**,**b**), as well as atomic force microscopy (AFM) profiles (**c**,**d**) and PL spectral variations (**e**,**f**). The numbered localized etch-pits (**a**) and the intersection of etched areas (**b**) obtained with enhanced spatial resolution are illustrated in top figures (**a**,**b**). The related optical images (bottom figures (**a**,**b**)) obtained in reflected light on the examined sample areas. The atomic force microscopy profiles scanned close to a single etch-pit (**c**) highlighted after short etching exposure; and rather extended areas (**d**) of the intersecting etched surfaces, formed under long etching procedures. The shape of the dislocation-ascribed single etch-pit can be employed for identification of dislocation type (as the edge dislocation ascribed etch pit illustrated in (**c**)). The deep etch-pits ascribed to either screw- or mixed type dislocations can be assumed by analyzing intersections of wide area etched hexagon valleys (**d**). The blue PL band spectral components appear only (**e**,**f**) when the confocal microscopy probe is localized either close to the core (location 2 in (**a**,**c**,**e**)—assuming dimensions of space charge region *R*_0_) of the single dislocation or steep planes of hexagonal valleys ((**b**,**d**,**e**), respectively). Numbers (1–8) denote locations nearby etch-pits where the PL spectra are recorded.

PL spectra in the etched samples were examined under pulsed UV excitation in a wide temperature range of 20–300 K. Evolution of these spectra as a function of 150 °C H_3_PO_4_ etching exposure is illustrated in Figure 2. The changes in spectrum structure and PL intensity were mainly obtained within blue PL bands when analyzing the samples covering the entire etching exposure range. Therefore, spectra ascribed to the initial and final stages of etch processing and the intermediate regime of 600 s exposure are illustrated in Figure 2. No surface pits could be resolved in the pristine sample (*t*_0_ = 0 s). Etching pits appeared after even a short etching duration (*t*_3_ = 600 s). Either cone- (*t*_3_) or hexagon (*t*_8_ = 1600 s)-shaped pits (Figure 2 and Figure 3c,d) appeared due to different rates of lateral and depth removal of material. The lateral dimensions of etching pits clearly increased with etching exposure when comparing the spatial extension of the etch-pit-profiles after *t*_3_ = 600 s and *t*_8_ = 1600 s (Figure 3) exposures.

It can be deduced from Figure 2d–f that the intensity of the violet PL peaking near 3.2 eV increased with the sample temperature in both pristine and etched samples. This outcome can be explained by an increase in excess carrier density with temperature, excited by UV laser light into conduction/valence bands; these carriers then recombine through donor-acceptor states. Only a weak short-wavelength B-PL wing appears on the background of YG-PL in non-etched samples when using pulsed excitation. The additional a B-PL band peaking at 2.7–2.9 eV appears in the etched samples starting from the shortest etching exposures (*t*_1_). This band has not been debated in detail in the literature. Intensity of the latter B-PL band increases with sample cooling and etching exposure. The intensity of the YG-PL also slightly increases with reduction in the sample temperature. The latter result can be understood by assuming variations in the concentration of the excess carriers recombining through the photoactive centers, as identified from photo-ionization spectroscopy. Correlation of the B-PL band intensity with dimensions of etching-pits, as an observed increase in B-PL peak amplitude with exposure to a fixed temperature (e.g., *T* = 20 K), implies a relation of these B-PL centers to dislocations. There, no 2.7–2.9 eV B-PL under pulsed UV excitation and no pits are observed for the pristine, non-etched samples. Meanwhile, B-PL appears, and its peak amplitude increases with exposure (*t*_3_) in the etched samples, in which etch-pits simultaneously manifest. Dimensions of etch-pits and the size of the hexagonal valleys (revealed by AFM scans, Figure 3c,d) also increase with etching duration.

The left column of Figure 3 illustrates a scanned profile of a rather bright pit, starting from the periphery of the 271 nm-wide and 65 nm-deep etch-pit. The peripheral zone (labelled as spectrum 1) and central area (spectra labelled as 3–5) exhibit PL spectra with a prevailing YG-PL band. There, a difference in spectral structure (a V-PL is lacking), relative to those recorded using UV pulsed laser (Figure 2d–f), appears due to peculiarities of the WITEC instrument. The WITEC scanner-spectrometer enables only room temperature measurements. On the other hand, the excitation density obtained using a sharply focused laser-diode beam in the WITEC system significantly exceeds that of a pulsed UV laser beam. Therefore, the intensity of the B-PL band peaking at 2.7–2.9 eV also becomes resolvable at room temperature using the WITEC scanner. Thereby, it can be inferred from the analysis of the evolution of PL topography that the B-PL band (within the spectrum labelled as 2 in the left column of Figure 3) appears when the excitation beam is localized at the boundary of the single etching-pit. This B-PL band is observable only for a narrow range (~10%) of etch-pit lateral dimensions. A long exposure time, which leads to the wide area and deeply etched material valleys, determines the YG-PL dominated spectral bands, though these bands spread out into the B-PL range (see the right column of Figure 3). Such a structure of spectral band, with the peak shifted toward short wavelengths, is better highlighted within the boundaries of the etched hexagons. This observation also implies that the B-PL peaking at 2.7–2.9 eV should be ascribed to the boundaries of etching pits.

Analysis of the shape of etching pits can be employed for rough identification of the dislocation type [21]. The triangle/cone-shaped etch-pit (left column, Figure 3) seems to be caused by edge dislocation. The hexagonal etch pits (right columns of Figure 2 and Figure 3) are inherent to screw dislocations. The wide area hexagonal valleys of etched GaN material containing local cone deeply imply mixed dislocations.

## 5. Discussion

The relations between photo-ionization (PI) and photo-luminescence (PL) spectra enable the prediction of a structure of Stokes-shifted PL bands on the basis of photoexcitation spectroscopy. Additionally, analysis of these correlations could serve for more reliable identification of the origin of the photoactive centers and their roles in the formation of PL bands.

In the literature, it has been widely reported that, within the photoluminescence spectra, the yellow-green (YG) band prevails in MOCVD GaN. This emission is probably carbon impurity related [10,44,45,46,47]. However, in earlier publications, this yellow luminescence band was alternatively associated with either gallium vacancy (V_Ga_) or a complex of gallium vacancy–oxygen on nitrogen sites (V_Ga_O_N_) [42,48,49,50,51]. On the other hand, either V_Ga_O_N_-2H or V_Ga_-3H complexes could be the reason for the appearance of yellow luminescence in highly vacancy rich samples, for instance, heavily irradiated GaN [10]. The carbon on nitrogen site (C_N_) defects or more complicated complexes composed of carbon on nitrogen site–oxygen on nitrogen sites (C_N_O_N_) can also cause the appearance of yellow luminescence (2.2 eV) in MOCVD GaN materials [10,45,46,52]. Therefore, it can be inferred that the YG band of GaN luminescence is composed of several spectral components attributed to different point defects. However, combining different excitation wavelengths indicates a prevalence of carbon attributed C_N_ and C_N_O_N_ complexes when 354 nm of excitation can efficiently stimulate electronic transitions within C_N_O_N_ complexes, while 408 nm of illumination activates the C_N_ centers [31]. Interstitial hydrogen is a widespread impurity in GaN crystal. It may interact with carbon-originated point defects, causing the appearance of blue luminescence. These hydrogen complexes with carbon-originated point defects seem to be non-homogeneously distributed blue luminescence centers that decorate the cores of dislocations. This B-PL is weak in non-etched samples at elevated temperatures when excess carriers diffuse out of the dislocation cores and B-PL light extraction is inefficient.

The presented characteristics of our experiments imply that the YG-PL band, peaking at 2.2 eV, is composed of PL processes ascribed to several E1–E4 photoactive centers, namely Ga vacancies (V_Ga_) and charged carbon impurities localized on N sites (C_N_^−^). This YG-PL band has a more sophisticated origin (relative to that ascribed to a single type defect [10]), as deduced from the correlation of photo-ionization and photo-luminescence spectra, compatible with comprehensive experimental results. The observed V-PL band, ascribed to radiative recombination through donor-acceptor pairs, can only be induced using UV excitation of excess carriers through inter-band transitions. The revealed B-PL in our experiments should be ascribed to point defect complexes decorating the cores of dislocations. This ascription is inferred from the appearance of the B-PL correlated with an etch-pit profile and the formation of a B-PL containing wide-PL bands at boundaries of deeply etched hexagonal valleys, as illustrated in Figure 3.

The evolution of a B-PL band with etching exposure can be explained by assuming local variations in PL light extraction efficiency when the area and geometry of cone or hexagon terrace boundaries are modified. As explained in [21], the rather high refractive index of GaN material determines the trapping of most B-PL photons generated in the vicinity of dislocation cores, due to total internal reflection. There is a very small critical angle between air and GaN due to the difference in the refractive index [21], and a relative area of the dislocation core of radius *R*_0_ = [*f*/π∆*a*(*N_D_* − *N_A_*)]^1/2^ [53] is considerably small for a non-etched GaN layer surface. Here, *f* is the Fermi distribution function; *N_D_* and *N_A_* are concentrations of donors and acceptors, respectively, which determine an extension of space-charge volume; and ∆*a* is a distance between broken bonds of the order of several angstroms for Si doped MOCVD GaN with *N_Si_* = 10^17^ cm^−3^, *R*_0_ ≤ 100 nm. This leads to the non-resolvable intensity of B-PL for non-etched GaN layers. The etching-pits appear due to different etching rates for horizontal *r_h_* and vertical *r_v_* removal of GaN material. The etch-pit depth *d* and width *l* are related to the etching exposure *t* as *d* = *r_v_t* and *l* = *r_h_t,* respectively, where it is always true that *l* > *d*. The etched surface provides more paths for B-PL light to escape from the space-charge region of dislocation cores. PL intensity *I_B-PL_*, measured as photon number *Φ* per time unit normalized to surface area, also increases due to the extraction of more photons via light scattering from the sidewalls of the etch pits. The etched terraces also have a larger surface area per unit volume crystal, from which B-PL is collected. However, defect complexes decorating the dislocation core constitute a *κ* fraction of the lattice atoms. The B-PL photons are scattered from the sidewalls of the etched cone with a side-surface area of 2π*ld.* The average number of recorded B-PL photons is proportional to a cone’s base area of *S* ≈ π(*r_h_t*/2)^2^. Thereby, the B-PL intensity is expressed as *I_B-PL_* ≈ Φπ(*r_h_t*/2)^2^ for a single etch-pit. Total intensity is also proportional to the dislocation density *D*. Thereby, *I_B-PL_* shows a parabolic increase with etch exposure, when the density of dislocations is invariable, and etch-pits do not coalesce. In the case of terrace formation through the merging of wide etch-pits, the total B-PL light collection area increases by partial component *A* of each (*m*) etching session-removed area. Then, *I_B-PL_* obeys the generalized dependence on etching exposure as follows:

(3) $$I_{B\text{-}PL}=\Phi Am\left[1+\left(\frac{\pi }{Am}\right){\left(\frac{r_{h}t}{2}\right)}^{2}\right].$$

The coefficient *M~*(*r_h_t*)^2^/*R*_0_ for total enhancement of *I_B-PL_* can be estimated using the ratio of light extraction areas *M* ≈ (*A/R*_0_) *=* (*π*/*R*_0_)(*r_h_t*/2)^2^|_for m=1_ when comparing these values in etched (~*t^2^*) and non-etched (~*R*_0_) samples. The dislocation decorating traps comprise only 0.1*R*_0_, as revealed from scans of B-PL constituent within the spectrum recorded on the periphery of etch-pits. The etched GaN surfaces might also increase the efficiency of PL excitation (due to enhancement of the excited area), resulting in a greater number of excess carriers and radiative recombination photons within etched samples.

**Figure 4:**
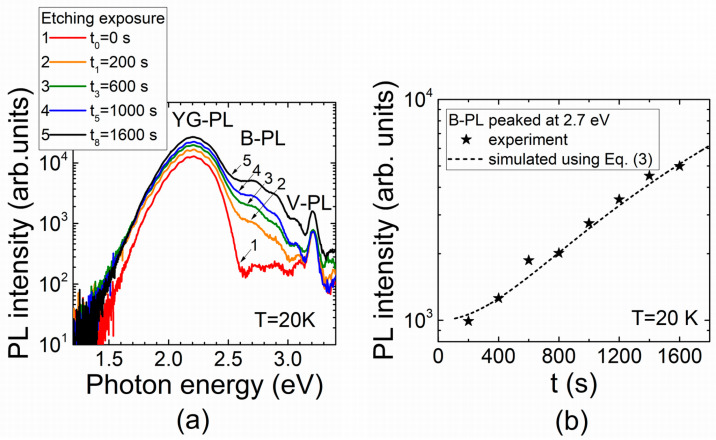
(**a**) Evolution of the PL spectra with H_3_PO_4_ etching exposure. (**b**) The B-PL band intensity (peaking at 2.7) evolution with etching exposure recorded (symbols) in MOCVD GaN using pulsed UV excitation and fitted (dashed curve) using Equation (3).

Variations in the PL spectral structure and the B-PL peak intensity dependent on etching exposure are illustrated in Figure 4. The *I_B-PL_* dependence on etching exposure, simulated using approximation Equation (3), is compared in Figure 4b with that measured at *T* = 20 K. This dependence indicates a nearly quadratic increase in the peak intensity after primary sessions of etching despite a later trend toward saturation appearing when the etched terrace area stabilizes.

**Figure 5:**
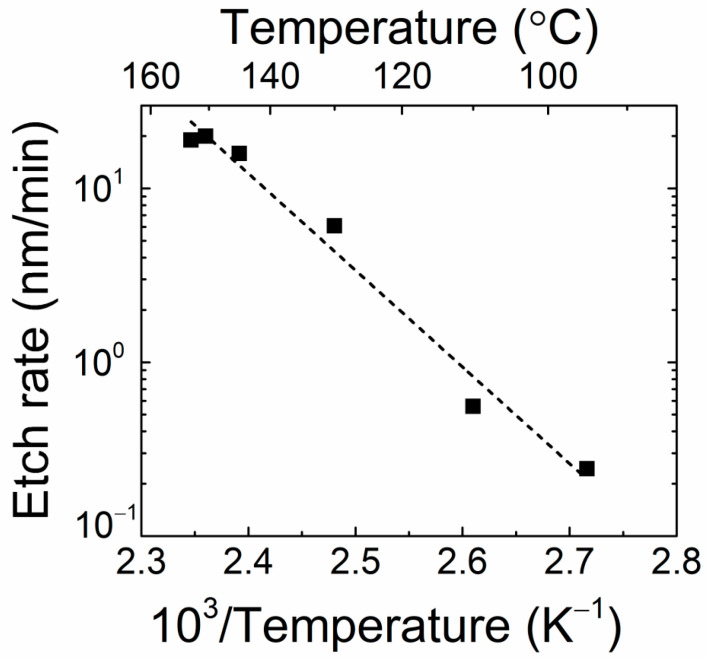
The horizontal etch rate as a function of H_3_PO_4_ acid temperature.

The horizontal etch rate *r_h_* additionally depends on H_3_PO_4_ temperature. The etching rate *r_h_* was evaluated using the relation *r_h_* = *l/t* and measurement data of lateral dimension *l* and exposure time *t*, extracted from etch-pit scans under the control of the acid temperature. This characteristic is illustrated in Figure 5.

It can be deduced from Figure 5 that fast removal of GaN material is achieved at elevated temperatures of 160 °C close to the boiling temperature of H_3_PO_4_, while a nearly exponential decrease in etching rate appears with the reduction in the acid temperature.

## 6. Summary

It has been found that red-shifted, relative to photo-ionization spectral peaks, bands of yellow-green luminescence (YG-PL) and blue (B-PL) luminescence are observed under pulsed UV excitation. The cross-correlations of the photo-ionization and photo-luminescence spectra, ascribed to the point photoactive centers, can be well simulated using the Kopylov–Pikhtin approach in the description of the absorption steps and vanRoosbroeck–Shockley relation to transform the photon-electron interaction cross-section data into photoemission spectra. It has been shown that this cross-correlation enables prediction of the Stokes shifts between the outspread PPIS steps and the respective PL band peaks. The recombination channel, ascribed to the violet PL band and peaking at 3.2–3.3 eV, corresponds to the donor-acceptor pair recombination. The additional B-PL band peaking at 2.7–2.9 eV has been revealed in the etched samples. The combination of different excitation wavelengths enabled the estimation of the prevailing PL centers. Carbon-oxygen C_N_O_N_ complexes prevail when 354 nm of excitation can efficiently stimulate electronic transitions within C_N_O_N_ defects, while 408 nm of illumination activates the C_N_ centers. The hydrogen complexes with carbon-originated point defects seem to be the non-homogeneously distributed blue luminescence centers, which decorate cores of dislocations. This B-PL is weak in non-etched samples at elevated temperatures when excess carriers diffuse out of dislocation cores, and B-PL light extraction is inefficient. The intensity of the latter B-PL band increases with sample cooling and etching exposure. Correlation of the B-PL band intensity with dimensions of etching-pits implies the relations of these B-PL centers to dislocations, where B-PL appears, and its peak amplitude increases with increased etching duration (*t*). The transforms of the etch-pit shape and dimensions simultaneously manifest with the increase in exposure. It has been inferred from the analysis of the evolution of PL topography that the B-PL band appears when the excitation beam is localized at the boundary of the single etching-pit, ascribed to a single dislocation. The wide area and deeply etched hexagon-shaped valleys appeared under prolonged etching exposures, followed by the formation of broad spectral bands spreading out into the B-PL range. The revealed B-PL could be ascribed to the point defect complexes decorating these cores of dislocations. This outcome has been deduced from the appearance of the B-PL correlated with the etch-pit profiles and formation of B-PL-containing wide PL bands at boundaries of deeply etched hexagonal valleys.

The evolution of the B-PL band with etching exposure has been explained by assuming local variations in PL light extraction efficiency when the area and geometry of cone or hexagon terrace boundaries are modified. The etching-pits appear due to different etching rates for horizontal *r_h_* and vertical *r_v_* removal of GaN material and based on scans of etch-pit dimensions dependent on acid temperature and etching exposure time *t*. It has been shown that the coefficient *M~*(*r_h_t*)^2^/*R*_0_ for the total enhancement of B-PL intensity can be estimated using the ratio of light extraction areas *M* ≈ *(π/R*_0_)(*r_h_t*/2)^2^ when comparing these values in etched (~*t*^2^) and non-etched (with radius *R*_0_ of dislocation core) samples. The etched GaN surfaces might also increase the efficiency of PL excitation (due to enhancement of the excited area), resulting in a greater number of excess carriers and radiative recombination photons within etched samples. A model of evolution of B-PL intensity with etching exposure has been approved.

## Figures and Tables

**Table 1:** Activation energy estimated by fitting PPI spectral steps recorded on MOCVD GaN and associated with different defects. Origin of these defects was identified using experimental activation energy values and theoretical parameters estimated based on configuration diagram models referenced in literature.

Photo-Active Center	Activation Energy (eV) ± 0.14 eV	*Γ*	Defect Type and Reference
E1	1.83	0.09	Unidentified [37]
E2	2.11	0.11	V_Ga_ [38]
E3	2.56	0.15	V_Ga_ [39]
E4	2.90	0.18	C_N_^−^ [31]
E5	3.04	0.15	C_N_^0^ [31]
E6	3.33	0.15	(C_N_O_N_)^0^ [31]

## Data Availability

Some of the data presented in this study are available on request from the corresponding author.
